# Pre-retirement Employees Experience Lasting Improvements in Resilience and Well-Being After Mindfulness-Based Stress Reduction

**DOI:** 10.3389/fpsyg.2021.699088

**Published:** 2021-07-15

**Authors:** Marina Diachenko, Kristina K. Smith, Lone Fjorback, Niels Viggo Hansen, Klaus Linkenkaer-Hansen, Karen Johanne Pallesen

**Affiliations:** ^1^Department of Integrative Neurophysiology, Center for Neurogenomics and Cognitive Research, (CNCR), Amsterdam, Neuroscience, VU Amsterdam, Amsterdam, Netherlands; ^2^Danish Center for Mindfulness, Department of Clinical Medicine, Aarhus University, Brabrand, Denmark

**Keywords:** stress reduction, resilience, resting-state thoughts and feelings, perceived stress, older employees, well-being, MBSR, mindfulness

## Abstract

The socio-economic benefits of interventions to prevent stress and related mental health problems are enormous. In the labor market, it is becoming desirable to keep employees for as long as possible. Since aging implies additional stressors such as increased risk of illness, and added pressure by professional tasks such as transferring knowledge, or learning new technologies, it is of particular relevance to offer stress-reduction to pre-retirement employees. Here, we report the effects of an eight-week Mindfulness-Based Stress Reduction (MBSR) intervention on mental well-being in 60–65-year-old work-active Danish employees, compared to a waiting-list control group. We observed improvements in resilience (Brief Resilience Scale) and mental well-being (WHO-5) not only at the end of the intervention, but also at the 12-month follow-up measurement that was preceded by monthly booster sessions. Interestingly, whereas well-being usually refers to experiences in the past weeks or months, we observed increasing Comfort in the MBSR-intervention group during a 5-minute eyes-closed rest session suggesting that this therapeutic effect of MBSR is measurable in how we feel even during short periods of time. We argue that MBSR is a cost-effective intervention suited for pre-retirement employees to cultivate resilience to prevent stress, feel more comfortable with themselves, maintain a healthy work-life in the last years before retirement, and, potentially, stay in their work-life a few more years than originally planned.

## Introduction

Work processes become more complex, more intense, and require flexibility and mobility from employees, now more than ever due to changes in the workforce spurred by globalization, digitization, and societal transformation (Mack et al., 2015). In addition to these demands that characterize the times we live in, employees approaching the end of work-life, typically around the age of 65, may experience additional stress, because aging implies its own sources of stressors such as a weakened defense to illnesses. Indeed perceived stress is an independent risk factor for illness and mortality (Prior et al., 2016). Long-term stress and associated feelings of control loss gives rise to psychological and physical diseases, such as heart disease, depression, type 2 diabetes (Stansfeld et al., 2002; Rosengren et al., 2004; Bartolomucci and Leopardi, 2009; Kelly and Ismail, 2015).

“Healthy aging” has been conceptualized as the sustained ability to adapt to the dynamic challenges of life (Juster et al., 2010), and hence relates closely to a well-regulated stress response, stress being defined as *an ongoing, adaptive process of assessing the environment, enabling the individual to anticipate and cope with changes and challenges* (McEwen and Akil, 2020). Despite additional stressors, imposed by aging, employees at pre-retirement age may wish to stay longer in the labor market because they find joy and meaning in their work and, importantly, workplaces may wish to hold on to the experience and wisdom offered by older employees—or society may decide to increase the retirement age. Retirement age has been steadily increasing since the 1990s in OECD countries (Denmark included) (Loichinger and Weber, 2016). As the proportion of older people in the population increases, there are calls for even further stimulation of labor force participation at older ages (Organization for Economic Co-operation and Development, 2006; United Nations Economic Commission for Europe, 2018). For the benefit of society and older employees it is important to help keep this cohort physically and mentally healthy in order to enjoy their last years in the workforce and be able to withstand the inevitable increase of the exit retirement age. Hence, identifying and implementing effective, evidence-based stress-management programs are urgently needed for pre-retirement employees.

The attempt to prevent and reduce perceived stress through strengthening our ability to adapt to changes and challenges is central in many self-development and mental training programs aimed at building resilience. A widely acknowledged prerequisite for adaptation is self-insight, especially awareness of personal strengths and weaknesses. One mental training program that has gained much support since its development in 1979 is Mindfulness-Based Stress Reduction (MBSR), which uses nonjudgmental attention training as its core feature in a body-oriented approach (yoga and meditation) combined with education on perception, stress biology, and communication (Kabat-Zinn, 1990). MBSR has been associated with a wide range of beneficial health effects that arise from the transformation of mental habits and behavioral schemes. Randomized controlled trials and meta-analyses of these show that MBSR training benefits individuals with or without a clinical diagnosis in terms of improvements on perceived stress, anxiety, depression, overall well-being, life satisfaction, and experienced quality of life (Khoury et al., 2015; Pallesen et al., 2016; Vibe et al., 2017; Juul et al., 2018, 2020; Young et al., 2018). In the MBSR exercises, attention is focused on the breath/bodily sensations, or stimuli in the immediate surroundings. The goal is to enhance awareness of sensations and feelings, such as signs of an overactivated stress response. Well-known to meditators, it is challenging to prioritize sensory presence over thoughts, which seem to constantly hi-jack our mind—a willful effort is needed to discipline attention. This training however—shifting between thoughts and sensations during the practice—raises the awareness of habitual thoughts that we tend to resort to and that often drive our feelings and related behavior. Gaining a meta perspective on our thought patterns enables a new perspective and an ability to regulate and transform maladaptive thoughts, feelings, and reactions into more adaptive ones that ultimately improve our mental and bodily health (Vago and Silbersweig, 2012).

In work places, the stress-reducing effects of mindfulness-based interventions are being measured as improved performance, productivity, agility, and innovative strength of organizations (Greiser and Martini, 2018). Studies show that workplace mindfulness interventions can lead to lessened emotional exhaustion potentially helping in preventing burnouts and more job satisfaction (Hülsheger et al., 2013). To our knowledge, no studies focused specifically on work-active older adults approaching retirement age. A number of studies address benefits of mindfulness training to retired older adults, showing beneficial effects on particular old-age-related health issues, such as insomnia (Zhang et al., 2015) and chronic back pain (Morone et al., 2008). It is plausible that mindfulness practice may promote healthy aging (Klimecki et al., 2019) by reducing symptoms of anxiety, depression, and stress, which are recognized risk factors for cognitive decline and dementia (Wilson et al., 2011; Diniz et al., 2013; Marchant and Howard, 2015; Gulpers et al., 2016). This association is corroborated by findings of improved memory and executive function in older adults following mindfulness training (Lenze et al., 2014; Moore et al., 2016; Wetherell et al., 2017).

The essential meaning of mindfulness is paying attention to sensations and stimuli in the present moment, hence associated with a decrease in mind wandering, i.e., attending to internally generated, stimulus-independent, thoughts and feelings (Rahl et al., 2017). Mind wandering is a strong mental propensity that occupies about half of our awake hours with impact on mood and task performance (Killingsworth and Gilbert, 2010; van Vugt and Broers, 2016; Irrmischer et al., 2018). Stressed individuals experience more mind-wandering and less engagement in/more rejection of the present moment (Crosswell et al., 2019). Generally, people feel more happy when focusing on the present (Killingsworth and Gilbert, 2010) and, conversely, psychiatric and neurological conditions are associated with an elevated tendency to mind wander (Christoff et al., 2016; Hoffmann et al., 2016). Not only the duration, but also the content of mind wandering can be quantified, and associated with mental health. Diaz et al. captured mind wandering—using the 5-min eyes-closed rest condition—and measured its content with the Amsterdam Resting-State Questionnaire (ARSQ) (Diaz et al., 2013, 2014). Using the ARSQ, reproducible patterns of individual thoughts and feelings have been observed, and associated with scores on insomnia, anxiety, and depression in large population samples (Diaz et al., 2013). Likewise, mental disorders including health anxiety, obsessive compulsive disorder (Gehrt et al., 2020) insomnia disorder (Palagini et al., 2016) and autism spectrum disorder (Simpraga et al., 2021) have been associated with distinct ARSQ profiles. Studies of the therapeutic benefits of MBSR have used clinical scales that refer to experiences during weeks or even months to assess participants’ well-being, as well as symptoms of stress, anxiety, and depression. Also sampling thoughts and feelings during 5-min rest could offer a new type of insight into MBSR participants’ state of mind. During the resting state, the mind typically wanders in a way that represents habitual ways of thinking, feeling and responding that MBSR targets and, thus, might be affected by the intervention. Hence, we apply the ARSQ to assess the effects of MBSR on these basic mental events.

In the present randomized control trial, we investigated the benefits of MBSR on mental health in 82 pre-retirement employees. All measures were sampled at three time points: before MBSR (T0), after MBSR (T4), and 12 months after the first measurement (T12). We used five validated questionnaires, which have been used previously as MBSR effect measures, namely, the Perceived Stress Scale (PSS), the Symptom Checklist 5 (SCL-5), the Satisfaction With Life Scale (SWLS), the Brief Resilience Scale (BRS), and the WHO-5 Well-Being Index. In addition, we used the ARSQ to sample thoughts and feelings during a 5-minute eyes-closed resting-state condition. We predict that MBSR leads to improvements in perceived stress (PSS), resilience (BRS), well-being (WHO-5), and symptoms of anxiety and depression (SCL-5). In addition, we predict that MBSR leads to changes in the resting state, measured with the ARSQ. In particular, we hypothesize increasing Comfort, and decreases in Discontinuity of Mind, Sleepiness, and Negative Thought.

## Materials and Methods

### Participants

Eighty-two healthy 60–65-year-old work-active employees in the private sector were recruited via public announcements on the website and social-media channels of the Danish Center for Mindfulness, Aarhus University. Exclusion criteria were a life-time diagnosis of psychosis, mania, or depression with psychotic symptoms.

### Experimental Design

After a series of open information meetings, persons who met inclusion criteria and were interested in participating signed up by booking a time for an individual interview at the Danish Center for Mindfulness in November-December 2019. They were given the opportunity to ask questions and if they wished to participate, they signed an informed consent form. Self-report data were collected in the REDCap electronic data collection and storage solution administered by Aarhus University, securing the protection of personal data (Harris et al., 2009). In the electronic questionnaires, which were administered at home, participants were guided through a 5-minute eyes-closed rest session followed by reporting of thoughts and feelings using the Amsterdam Resting-State Questionnaire (ARSQ), after which they filled in five validated questionnaires: the Perceived Stress Scale (PSS), the Brief Resilience Scale (BRS), the WHO-5 well-being scale (WHO-5), the Symptom Checklist-5 (SCL-5), and the Satisfaction With Life Scale (SWLS).

**FIGURE 1:**
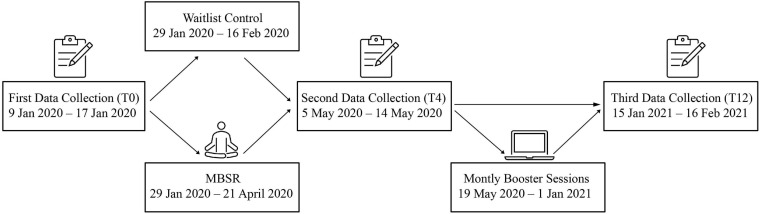
Timeline of study. After fist data collection (T0), a randomization procedure allocated the participants into either the MBSR intervention group, receiving the 8-weeks MBSR intervention, or the waiting-list control group. The MBSR intervention was halfway transformed to an online intervention with monthly booster sessions preceding shortly after. All participants had their data collected for a second time (T4) and again a third time (T12). MBSR: Mindfulness-Based Stress Reduction.

**FIGURE 2:**
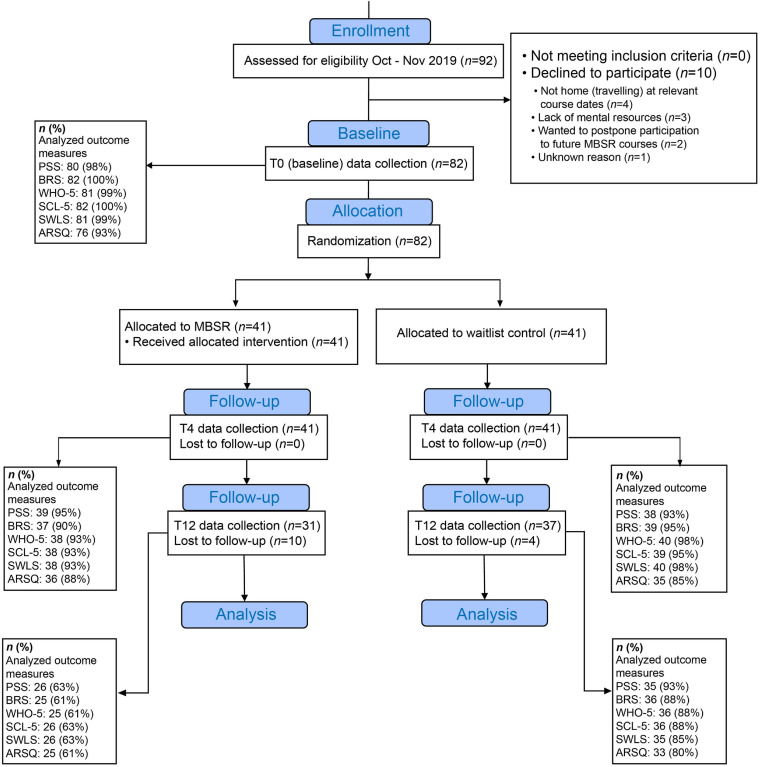
Flowchart of study design. Due to the COVID-19 pandemic, the original experimental design was modified from a 2-year multi-method study to a 1-year study based on self-report electronic questionnaires data. Participant recruitment stalled due to the first COVID-19 lockdown in March 2020, and the number of participants was reduced from the planned 192 to 82. The first MBSR course, which was then halfway, was transformed to an online intervention with monthly booster sessions. Originally planned collection of physiological data at T4, and T12 eventually had to be abandoned. The study was rounded up prematurely in January 2021. T0: baseline measurement. T4 and T12: measurements after 4 and 12 months, respectively. MBSR: Mindfulness-Based Stress Reduction. PSS: Perceived Stress Scale. BRS: Brief Resilience Scale. WHO-5: the well-being scale. SCL-5: Symptom Checklist-5. SWLS: Satisfaction With Life Scale. ARSQ: Amsterdam Resting-State Questionnaire.

When all participants had completed the T0 questionnaire, an automatized randomization procedure allocated them into either: (1) the MBSR intervention group, receiving the 8-weeks MBSR intervention a few weeks later, or (2) the waiting-list control group that received the MBSR after T12 data collection had been completed. An independent data manager had programmed the randomization algorithm in REDCap. Of the 82 participants, 41 were allocated to the MBSR-intervention group (28 females, 13 males) and 41 to the Control group (24 females, 17 males). The timeline of the three data collections (T0, T4, T12) and the MBSR intervention is illustrated in Figure 1. The study design, participant compliance and questionnaire completion are illustrated in the flow chart in Figure 2. The study was conducted according to the Helsinki Declaration (World Medical Association, 2000). It was approved by the Danish Research Ethics Committee. All participants were informed verbally and in writing and signed a written informed consent form prior to participation.

### MBSR Intervention

The Mindfulness Based Stress Reduction (MBSR) intervention was delivered by an experienced MBSR teacher trained at the Danish Center for Mindfulness. MBSR is a standardized curriculum-based program in which participants learn a set of mental and physical training methods (relaxation, meditation, yoga) and receive materials (sound files with guided exercises) to enable training at home. The standard delivery of MBSR is a series of group sessions, eight weekly 2.5 h sessions and a full-day (7 h) session (UMass Memorial Health Care, 2019). Adherence to the home exercises during the MBSR program has been positively related to the extent of improvement in several measures of symptoms and well-being (Carmody and Baer, 2008; Parsons et al., 2017). Therefore, before starting the course, participants are told that it involves a home training volume of 45 min/day during the course. Participants are encouraged to continue this volume of training after the course, because just like with physical health, in order to uphold the benefits, ongoing training is needed.

After 5 regular MBSR group sessions, due to COVID-19-related imposed restrictions in mobility, the remainder of the course was completed in the form of online sessions. Based on general participant feedback we deem that the character and total intensity of the intervention was conserved in spite of this transition. In addition, as a consequence of COVID-19, it was decided to develop and offer a series of monthly online follow-up “booster” MBSR sessions to support the continued practice for the participants in the MBSR-intervention group. This was originally intended to bridge the time period until the original protocol’s post-intervention physiological follow-up data collection procedures could be made, but in the course of the prolonged lockdown the booster sessions became a spin-off with its own significant value to some participants—many of whom were otherwise socially quite isolated. This led to the continuation of online booster sessions for 10 months. The monthly booster sessions were 2 h long and optional, with about a third of MBSR-group participants joining each time. The content of the booster sessions was similar to the weekly meetings in the MBSR program, with guided meditations, articulation exercises and group dialogues, with a series of themes that reflected participants’ experiences related to work/life balance, the COVID-19 crisis, retirement plans, etc. — as well as continuing and deepening mindfulness practice.

The waiting-list group received no particular instructions. They were encouraged to continue life as usual, even if this involved some mindfulness practices or similar.

### Outcomes

Based on the extensive literature on the positive effects of MBSR on stress and well-being, we chose five validated clinical scales covering these domains (Juul et al., 2020). The BRS was included in these scales. Although measures of resilience were previously not extensively applied in mindfulness studies, it is a reasonable assumption that the stress-reducing effects of MBSR implicate measurable enhancements in resilience, previously indicated in e.g., one of our own studies (Juul et al., 2020) and another study (Nila et al., 2016). The ARSQ was included to test the applicability of 5-min samples of thoughts and feelings to capture expected changes in mental dimensions related to changes in the five classic questionnaires.

### Primary Outcome

#### The Perceived Stress Scale (PSS)

The PSS is a self-report measure of subjective stress (Cohen et al., 1983). It consists of 10 questions indicating how often respondents have found their life unpredictable, uncontrollable, and overloaded in the past month. All items are scored on a five-point Likert scale from 0–4 (0 = never, 4 = very often) (total sum scores: 0–40). A score of 0–13 indicates low perceived stress, 14–26 moderate perceived stress, and 27–40 high perceived stress. The PSS has demonstrated good validity and reliability (Cohen et al., 1983; Lee, 2012; Eskildsen et al., 2015). A population-based study has shown a dose-response relationship between perceived stress measured by the PSS and mortality within a four-year period (Prior et al., 2016).

### Secondary Outcomes

#### The Brief Resilience Scale (BRS)

The BRS is a self-report measure of resilience that inquiries about the respondent’s perceived ability to bounce back/recover from stress (Smith et al., 2008). The scale contains six statements, which are rated on a Likert scale from 1–5 (1 = strongly disagree, 5 = strongly agree), and the summary score is the average of the six items (range 1–5) (Smith et al., 2008). The following cut-off points have been suggested: Scores from 1.00–2.99: low resilience; 3.00–4.30: normal resilience; 4.31–5.00: high resilience.

#### The WHO-5 Well-Being Scale (WHO-5)

The WHO-5 is a self-report measure of well-being (World Health Organisation, 1998). The respondent is asked to rate five statements on a 6-point Likert scale from 0–5 (0 = at no time, 5 = all the time). Each question assesses how often respondents have experienced specific positive thoughts or feelings in the past two weeks. The points are added and multiplied with four, calculating the total score ranging from 0–100; higher scores indicate a higher level of well-being. The WHO-5 well-being scale is considered to be a valid measure of the overall well-being with scores lower than 52% (corresponding to raw score = 13) considered critically low (Topp et al., 2015).

#### The Symptom Checklist-5 (SCL-5)

The SCL-5 is a self-report questionnaire to assess psychological distress, and symptoms of anxiety and depression (Tambs and Moum, 1993). The scale refers to the last 2 weeks and consists of five statements that are scored on a scale from 1 (not at all) to 4 (very much). The score is calculated as the average of the five items with higher scores indicating greater symptoms of anxiety and depression. The SCL-5 originates from the 25-item Symptom Checklist (SCL), which has been applied to detect mental disorders (Joukamaa et al., 1994) and correlates at *r* = 0.92 with the SCL. An SCL-5 score > 2 has been found to predict the presence of a mental illness, as assessed independently by psychiatrists (Strand et al., 2003).

#### The Satisfaction With Life Scale (SWLS)

The SWLS is a self-report questionnaire that measures global cognitive judgments of satisfaction with one’s life (Diener et al., 1985). The scale consists of five statements that are rated on a 7-point Likert scale from 1 (strongly disagree) to 7 (strongly agree). The total score is 5–35; higher scores indicate greater satisfaction with life with scores of 5–9 being “extremely dissatisfied”, 10–14 “dissatisfied”, 15–19 “slightly dissatisfied”, 20 “neutral”, 21–25 “slightly satisfied”, 26–30 “satisfied”, and 31–35 “extremely satisfied”. SWLS has demonstrated high validity and reliability (Pavot et al., 1991; Howell et al., 2010).

#### The Amsterdam Resting-State Questionnaire (ARSQ)

**FIGURE 3:**
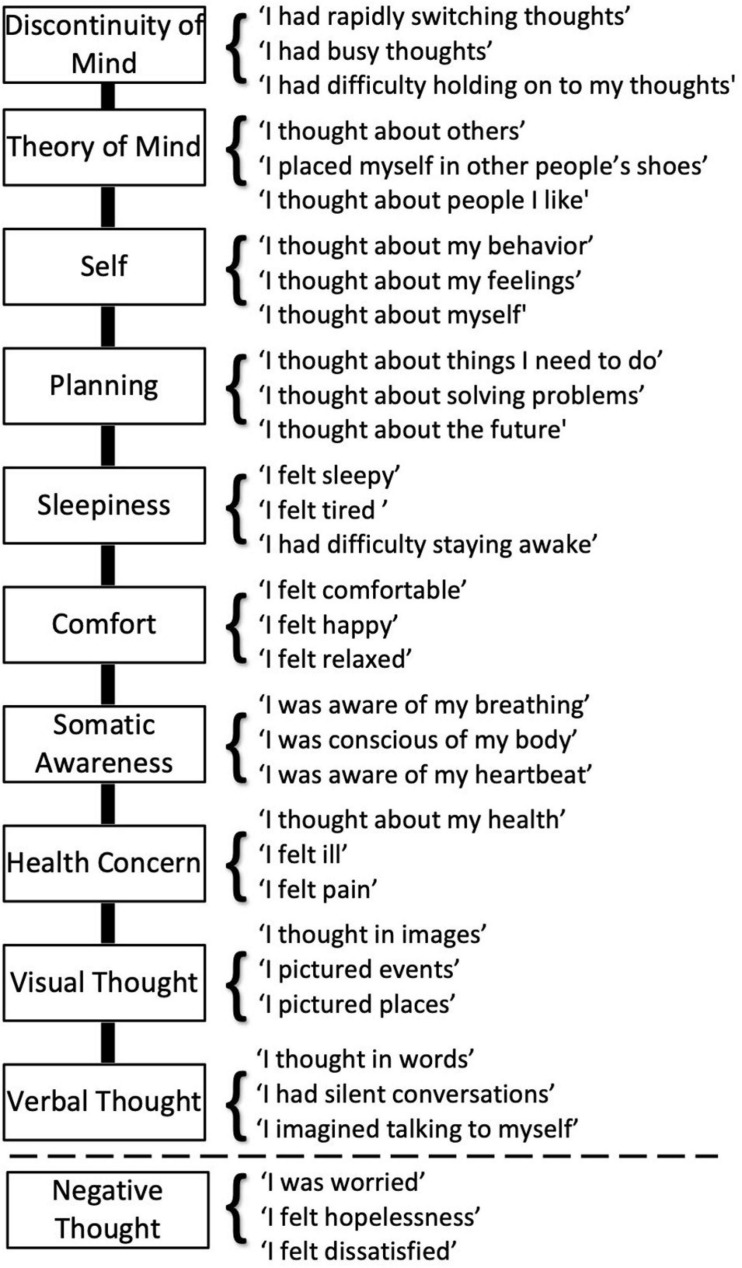
An extended Amsterdam Resting-State Questionnaire capturing eleven dimensions of thoughts and feelings. The ARSQ 2.0 model of mind-wandering contains 10 factors defined by the items listed above the dashed line (Diaz et al., 2014). For the present study, we added the dimension labelled “Negative Thought”.

The ARSQ is a self-report measure of the content and quality of thoughts and feelings experienced during a resting state (Diaz et al., 2013). The ARSQ identifies 10 dimensions: Discontinuity of Mind, Theory of Mind, Self, Planning, Sleepiness, Comfort, Somatic Awareness, Health Concern, Visual Thought, and Verbal Thought. In the present study, we extended the 10-dimensional model of the ARSQ 2.0 (Diaz et al., 2014) with an experimental dimension labelled Negative Thought (Figure 3). The score on each of the 11 dimensions was calculated as the mean score of three items that were rated on a five-point ordinal scale (1 to 5) corresponding to the labels “Completely Disagree,” “Disagree,” “Neither Agree nor Disagree,” “Agree,” and “Completely Agree.”

#### Other Questions Asked

In the online questionnaire, we also asked a number of additional questions about participants’ previous experience with yoga and/or meditation. *“Do you have previous experience with yoga and/or meditation?”* (“yes” or “no”) and (2) “*How often do you practice yoga and/or meditation?”* (“every day”, “every week”, “every month”, “rarely”, or “never”, corresponding to a 0–4 scale). We also inquired about participants’ engagement in the home exercises that constitute an important part of the MBSR program (see Materials and Methods). The question was phrased like this: *“During the last week, how many times have you done yoga or meditation exercises (e.g., from the MBSR program or other sources)?”.* We also asked the participants of the MBSR group to give an estimate of their adherence to the booster sessions: *“How many booster sessions have you attended?”* (“none”, “a few”, “most” or “all”). Finally, we also asked participants to rate their current sleep quality as “very poor”, “poor”, “mixed”, “good”, or “very good” (corresponding to a 1–5 scale). We did this to acknowledge that sleep quality plays a central role to health in general, and that sleep disturbances pose a significant medical and public health concern for the aging population (Black et al., 2015). For example, an estimated 50% of people 55 years and older have problems initiating and maintaining sleep (Van Cauter et al., 2000; Foley et al., 2004). We also asked participants to rate their experienced job satisfaction as: very unsatisfied, dissatisfied, neither satisfied nor dissatisfied, satisfied, or very satisfied (corresponding to a 1–5 scale), and to report illness-related absence: *“How many days were you absent from work the last month?”*.

### Statistical Analysis

#### Linear Mixed-Effects Model

Linear mixed-effects models (LMMs) offer a statistical framework to analyze unbalanced longitudinal data structures with covariance among the repeated measures allowing to model both between- and within-subject sources of variability (Fitzmaurice et al., 2011). We used LMM to model the changes in the mean response for self-reported cognitive outcomes and test the null hypotheses of no intervention effect on changes in the mean response over time between the Control and MBSR-intervention group. Time, group, and their interaction were specified as fixed effects, and random intercepts on subject level were added to the model to allow for subject-specific idiosyncrasies in their propensity to respond. If *N* is the total number of subjects and *n_i* is the number of measurement occasions for subject *i* (*i* = 1,…,*N*), then the model can be expressed as following:

$$Y_{ij}=\beta_{0}X_{ij0}+\beta_{1}X_{ij1}+\beta_{2,j}X_{ij2}+\beta_{3,j}X_{ij1}X_{ij2}+b_{i}+e_{ij},j=1,n_{i},$$

where *Y*_*ij*_ = (*Y*_*i*1_,…,*Y*_*in*_*i*__)^*T*^ are subject responses, β_*j*_ = (β_0_,β_1_,β_2,*j*_,β_3,*j*_)^*T*^ are population regression parameters (fixed effects), *e*_*ij*_ = (*e*_*i*1_,…,*e*_*in*_*i*__)^*T*^ are measurement errors *b*_*i*_ = (*b*_1_,…,*b*_*N*_) are subject random effects (*e* and *b* are assumed to be independent and have zero-centered normal distributions), and *X*_*ij*_ = (*X*_*ij*0_,…*X*_*ij*2_)^*T*^ are predictors, with *X*_*ij*0_ = 1 for all *i* and *j*, *X*_*ij1*_ representing the Control or MBSR-intervention group, and *X*_*ij2*_ – the month in which the responses were collected. In our study, *N=82* subjects, *n_i= 3* measurements: pre-intervention (*T0*), post-intervention after four months (*T4*), and at follow-up after twelve months (*T12*). As an example, *Y*_*ij*_ could be the score of subject *i* at measurement occasion *j* on the Brief Resilience Scale.

With this model, we compared the mean response profiles over time between the Control and MBSR-intervention group, μ_*j*_(*Con*) and μ_*j*_(*MBSR*), respectively. The null hypothesis is that the change in mean over time does not differ between the two groups at either measurement occasion:

$$H_{0j}:\mu_{j}\left(MBSR\right)-\mu_{T0}\left(MBSR\right)=\mu_{j}\left(Con\right)-\mu_{T0}\left(Con\right),j=T4,T12.$$

This can also be expressed in terms of the regression coefficients β. For that, we assign *X*_*ij*1_ = 0 to the Control and *X*_*ij*1_ = 1 to the MBSR-intervention group. Then, the mean response profile for the Control group can be written as:

$$\mu_{j}\left(Con\right)=E\left(Y_{ij}|X_{ij}\right)=\beta_{0}+\beta_{2,j}X_{ij2},j=T0,T4,T12,$$

where *X*_*ij*2_ = 0 for the baseline measurement (*j* = *T*0), and *X*_*ij*2_ = 1 for *j* = *T*4,*T*12. The mean response profile for the MBSR-intervention group will be:

$$\mu_{j}\left(t\right)=E\left(Y_{ij}|X_{ij}\right)=\left(\beta_{0}+\beta_{1}\right)+\left(\beta_{2,j}+\beta_{3,j}\right)X_{ij2},j=T0,T4,T12.$$

Coefficient β_0_ is the intercept and corresponds to the mean response of the Control group at *T0*, β_1_ is the shift from the intercept for the MBSR-intervention group at *T0*, β_2_ is the slope for the Control group (the change in mean from *T0* to *T4* or *T12*), and β_3_ is the shift from the slope for the MBSR-intervention group (i.e., how much the change in mean of the MBSR-intervention group from *T0* to *T4* or *T12* is different from that of the Control group). Now, the null hypothesis is that the change in mean for the MBSR-intervention group is not different from the change in mean for the Control group:

$$H_{0j}:\left(\beta_{0}+\beta_{1}\right)+\left(\beta_{2,j}+\beta_{3,j}\right)-\left(\beta_{0}+\beta_{1}\right)=\beta_{0}+\beta_{2,j}-\beta_{0},j=T4,T12,$$

which simplifies to testing whether the difference between the change in mean of the MBSR-intervention group and the one of the Control group is not different from zero:

$$H_{0j}:\beta_{3,j}=0,j=T4,T12$$

For each outcome variable, we fitted a linear mixed-effects model by Maximum Restricted Maximum Likelihood (REML) using *lmerTest* package (Kuznetsova et al., 2017) with Satterthwaite’s approximation (Luke, 2017) for degrees of freedom, *t*-statistics, and *p*-values for the fitted regression coefficients in R (R Core Team, 2019). A two-sided significance level of 0.05 was used for hypothesis testing. Due to the exploratory nature of the analysis, no multiple testing *p*-value adjustments were made (Feise, 2002; Althouse, 2016; Parker and Weir, 2020). Residuals were explored to evaluate the model’s statistical assumptions (i.e., normality, homogeneity of variance) (Fitzmaurice et al., 2011).

Data were analyzed using MATLAB, 2020 (The MathWorks Inc., Natick, MA, United States) and R (R Core Team, 2019, Vienna, Austria). Due to missing data points, a few datasets were incomplete so that the number of participants varied slightly for different outcome variables at T0, T4, or T12 (see Figure 2). Only missing measurements per time point were dropped, allowing to retain the remaining data for each participant. We included all available data because the linear mixed model statistical framework can handle missing data and including all data maximizes the statistical power. For example, it would be unfortunate to reduce statistical power of the T4 vs. T0 comparison by excluding participants that dropped out at T12.

## Results

To investigate the potential benefits of mindfulness training on the mental health of pre-retirement employees, we performed a randomized waitlist-controlled trial on 82 (males = 37%, females = 63%) 60–65-year-old workers from the private sector (for details, see Materials and Methods). Self-report measures of mental health and thoughts and feelings experienced during 5-minutes wakeful rest were collected at baseline (T0), and after 4 and 12 months (T4 and T12) (Figure 2).

### Baseline Scores

The mean baseline scores on the five psychometric measures at the beginning of the study (T0) provided indicators of the total group of participants’ mental health status. Most mean scores were within established normal ranges (mean ± standard deviation: PSS = 15.4 ± 7.0, BRS = 3.5 ± 0.7, WHO-5 = 62.2 ± 20.4, SCL-5 = 1.8 ± 0.6, and SWLS = 25.3 ± 6.4). The PSS ratings indicate a high prevalence of stress with 46% of participants falling in the “moderate stress” range (14–26) and 10% falling in the high-level stress range (27–40), which are stress levels that have been associated with both physical and mental impairments (Prior et al., 2016). The average resilience score (3.5 ± 0.7) was in the low end of “normal” (3.00–4.30), and the ratings from 22% of the 82 participants corresponded to “low resilience” (1.00–2.99) at baseline. Twenty-five percent of participants scored below the cut point WHO-5 score of 54, which is considered a threshold for poor well-being. Similarly, 26% of the participants scored above the SCL-5 cut-point of 2, which predicts the presence of mental illness. The average SWLS score (25.3 ± 6.4) falls in the high end of the category “slightly satisfied”, hence above “neutral,” while 21% of the participants fall in the category “neutral” or below. There was no significant difference in means between the two groups at T0 for each of the five psychometric measures (Supplementary Table 1).

**FIGURE 4:**
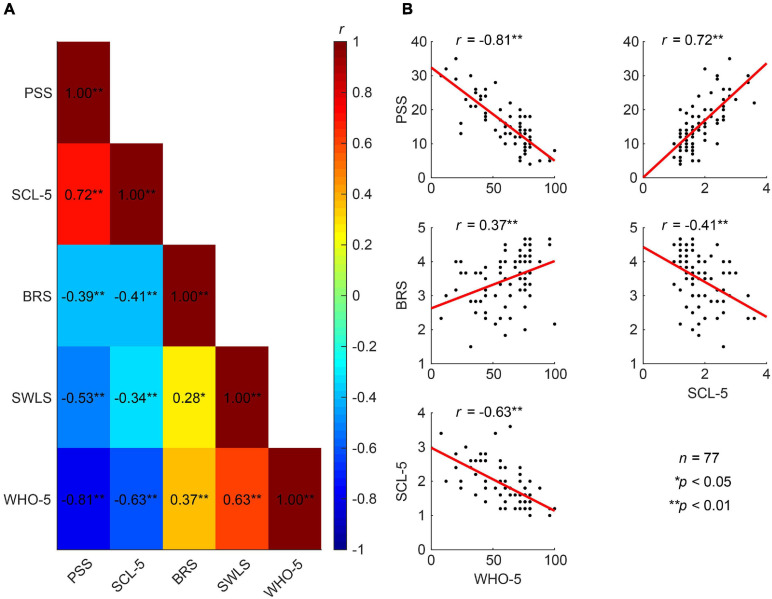
Correlations between different measures of mental well-being. **(A)** Correlation heat map between the five established psychometric scales related to mental health (Pearson correlation coefficients). **(B)** Well-being (WHO-5) exhibits strong negative correlations with perceived stress (PSS) and symptoms of anxiety and depression (SCL-5), and a moderate positive correlation with resilience (BRS). The SCL-5 also shows strong positive correlations with PSS and negative correlations with the BRS.

We further used the baseline data (T0) of the five psychometric measures from the total cohort to investigate how the measures relate to one another. Albeit the statements asked in the different self-report questionnaires are very different, they all tap into different aspects of mental well-being and, thus, show moderate to strong correlations (Figure 4).

### Effects of MBSR on Perceived Stress (PSS), Resilience (BRS), Well-Being (WHO-5), Symptoms of Anxiety and Depression (SCL-5), and Satisfaction With Life (SWLS)

In order to assess the effect of the MBSR training at T4 and T12, we fitted a linear mixed model (LMM) to the five questionnaires *PSS, BRS, WHO-5, SCL-5 and SWLS*. Specifically, we compared the change in mean for the MBRS-intervention group with that of the Control group, which is reflected in the b_3_-coefficient of the LMM analyses (Materials and Methods).

**FIGURE 5:**
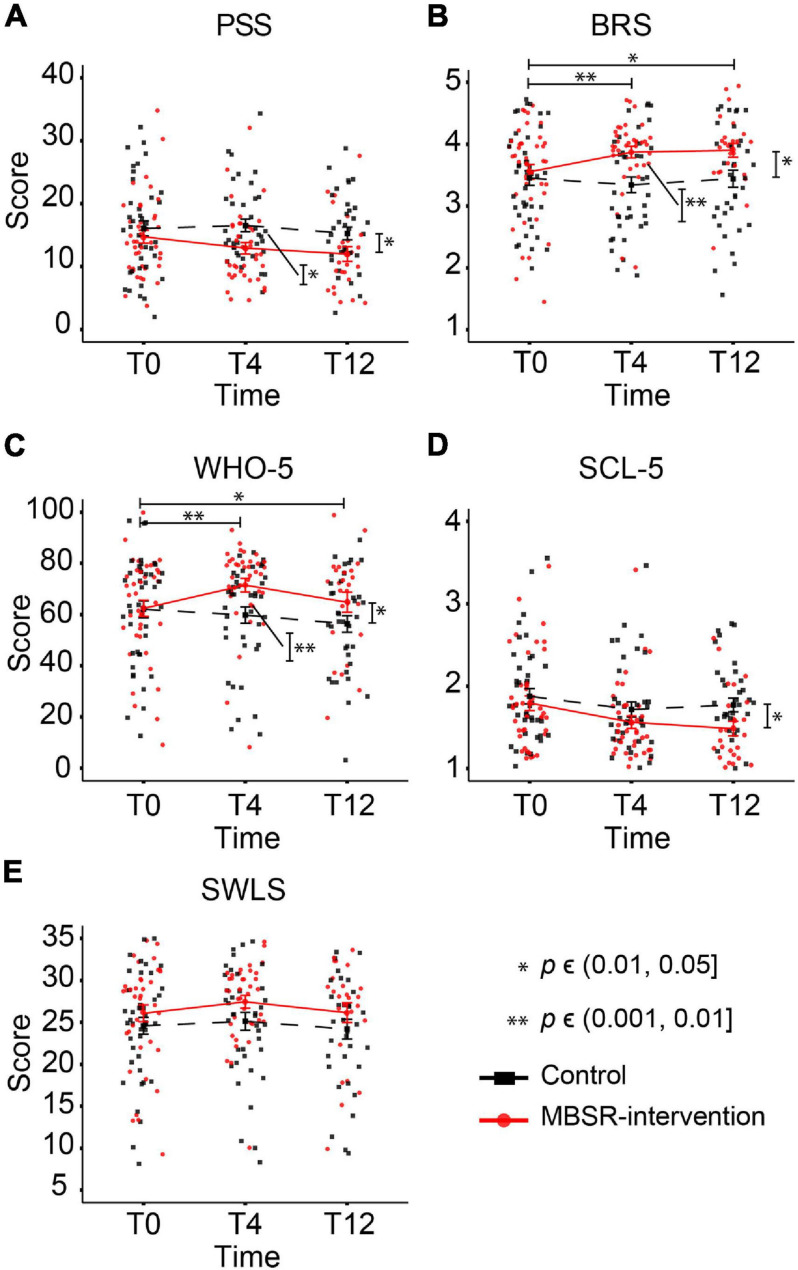
The MBSR-intervention group shows superior and lasting improvements in resilience and well-being compared to the Control group. The individual participant scores and the group-mean scores are shown for each of the five psychometric measures: PSS **(A)**, BRS **(B)**, WHO-5 **(C)**, SCL-5 **(D)**, and SWLS **(E)**. Significant differences in the estimated changes over time in the two groups are shown with horizontal bars and asterisks. Vertical bars and asterisks indicate a significant difference between the group sampling means at each individual time point. All statistics are based on LMM, see Table 1 for details. T0: baseline measurement. T4 and T12: measurements after 4 and 12 months, respectively.

We did not observe a significant superior difference in perceived stress, the primary outcome variable of this study (PSS mean change difference −1.94, 95% CI = [−4.52, 0.62], *p* = 0.143, Table 1). Nevertheless, we saw a significant reduction in sample-mean PSS scores in the MBSR-intervention group compared to those in the Control group both at T4 (sample-mean difference of PSS = −3.61, 95% CI = [−6.00, −0.42], *p* = 0.026) and at T12 (PSS = −5.26, 95% CI = [−6.08, −0.05], *p* = 0.049) (Table 1 and Figure 5A). The MBSR group showed superior improvements to the Control group in resilience (BRS mean change difference = 0.40, 95% CI = [0.16, 0.65], *p* = 0.002) and well-being (WHO-5 mean change difference = 10.90, 95% CI = [2.86, 18.95], *p* = 0.009) after four months (Figures 5B,C). Albeit these effects were less pronounced after 12 months, they remained significant (BRS mean change difference = 0.27, 95% CI = [0.01, 0.54], *p* = 0.049; WHO-5 mean change difference = 9.89, 95% CI = [1.02, 18.74], *p* = 0.031). Importantly, the BRS and WHO-5 showed no significant group differences at baseline (T0, Table 1) and no significant differences in the means within groups between T4 and T12, which indicates that MBSR had positive effects far beyond the intensive 8-week training (Table 1).

**TABLE 1:** Linear mixed model analyses show superior and lasting improvements in resilience and well-being for the MBSR-intervention group compared to the Control group.

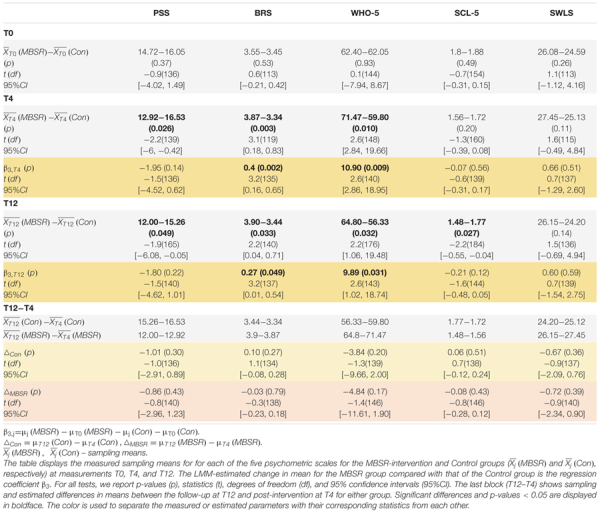

The LMM analysis did not reveal significant effects on SCL-5; nonetheless, the MBSR participants scored lower on the symptoms checklist than Controls both at T4 and T12, reaching statistical significance in sample means difference at T12 (Figure 5D and Table 1). Before MBSR training, participants in the MBSR group had an average score of 1.8, which decreased to 1.56 at T4 and 1.48 at T12. In comparison, the control group was at 1.88 (T0), 1.72 (T4) and 1.77 (T12). Looking closer at the development around the SCL-5 cut point of 2.0, we note that while the percentage of participants in the MBSR group with a score above 2 changes from 22% (T0) to 11% (T4) to 12% (T12), the analogous numbers in the Control group are 29% (T0), 23% (T4), and 22% (T12), suggesting that especially participants with high levels of symptoms were helped. Similarly, the MBSR group scored higher on satisfaction with life (SWLS) compared to the Control group but this effect did not reach significance (Figure 5E and Table 1). In general, group differences between the baseline scores were nonsignificant for each of the five psychometric measures (see block T0 in Table 1), with no significant changes in means within the control group between T0 and T4 or T0 and T12 (see b_2_-coefficients of the LMM in Supplementary Table 1). For a complete report of the LMM analysis, see Supplementary Table 1.

### Previous Experience, Home Practice and Booster Session Participation

Amid factors that might have affected the observed effect of MBSR was participants’ previous experience with yoga and meditation. Thus, we looked into differences between the two categories of participants—with and without previous practice—in the MBSR-intervention group. Overall, 25 participants in the MBSR group indicated to have had previous experience and 16 to not have any experience. Of note, four out of the five scales indicated that MBSR participants with no previous practice presented with significantly better mental well-being at baseline (T0 in Table 2) compared to MBSR participants with experience, i.e., symptoms of anxiety and depression were lower, whereas scores on resilience, satisfaction with life, and well-being were higher in MBSR participants without previous experience (sample-mean difference of PSS = −1.69, 95% CI = [−5.49, 2.1], *p* = 0.391; SCL = −0.34, 95% CI = [−0.65, −0.03], *p* = 0.035; BRS = 0.75, 95% CI = [0.38, 1.12], *p* < .001; SWLS = 5.84, 95% CI = [2.55, 9.12], *p* = 0.001; WHO-5 = 15.97, 95% CI = [4.89, 27.05], *p* = 0.007). After the intervention (block T4 in Table 2), the scores improved on each of the five psychometric outcomes in both categories of participants, except a slight decrease on SWLS for participants without experience. Overall, the improvements were greater for participants with previous yoga/meditation experience, both at T4 and T12. For a complete report of the LMM analysis, see Supplementary Table 2.

**TABLE 2:** The MBSR-intervention participants with previous yoga/meditation experience show significantly poorer baseline scores on BRS, SCL-5, SWLS, and WHO-5 scores as compared to MBSR-intervention participants without previous experience and improve on these psychometric outcomes after the intervention.

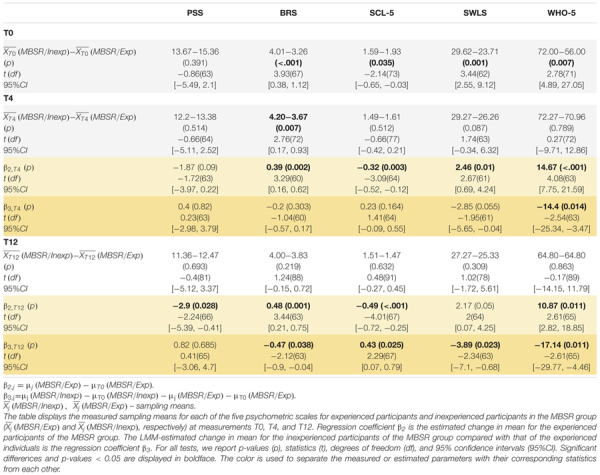

To further gauge relevant participant behavior and experienced benefits, we looked at the frequency of yoga or meditation home practice sessions during the preceding week reported by all the participants at T4 and T12. At the follow-up after four months, participants in the MBSR group reported to have exercised approximately 4 times on average during the previous week (mean ± SD: 3.9 ± 3.6), which dropped to 2.7 times after twelve months (2.7 ± 3.5, t-test *p*-value < 0.001, Cohen’s ds = 0.89). As expected, the Control group engaged in less home meditation and yoga practice than the MBSR group both at T4 and T12 (1.2 ± 2.4 and 1.0 ± 1.8, respectively). Our inquiry about participation in MBSR-booster sessions revealed that 2% (*n* = 1) participated in all sessions, 27% (*n* = 11) of participants took part in most sessions, 37% (*n* = 15) in some, and 5% (*n* = 2) did not attend any booster sessions provided to the MBSR group. Twenty-nine percent of participants (*n* = 12) did not provide an answer. The participants did not show a clear preference for the session format: 41% favored physical meetings, 24% preferred online sessions, and 35% preferred a mixed setup.

### Sick Days, Job Satisfaction, and Sleep Quality

In spite of approximately a quarter of participants scoring in a critical range on the clinical scales at baseline, absence due to sickness was very low with 78% reporting zero sick days in the past month in both groups. This number increased 20 percentage points at T4 in the MBSR group (to 98%) and 5 percentage points in controls. At T12, only 10% (MBSR) and 11% (Controls) reported any sick days. Job satisfaction was generally high with mean scores close to 4 (“satisfied”) for both groups throughout the study and no significant intervention effects. MBSR participants reported a significant improvement in sleep quality from T0 to T4, compared to the Controls (Sleep quality mean change difference 0.32, 95% CI = [0.04, 0.6], *p* = 0.03). For a complete report of the LMM analysis of Sleep quality and job satisfaction, see Supplementary Table 3.

### A 5-Minute Sample of Resting-State Thoughts and Feelings Correlates With Self-Reported Mental Health—and Can Be Modulated With MBSR

**FIGURE 6:**
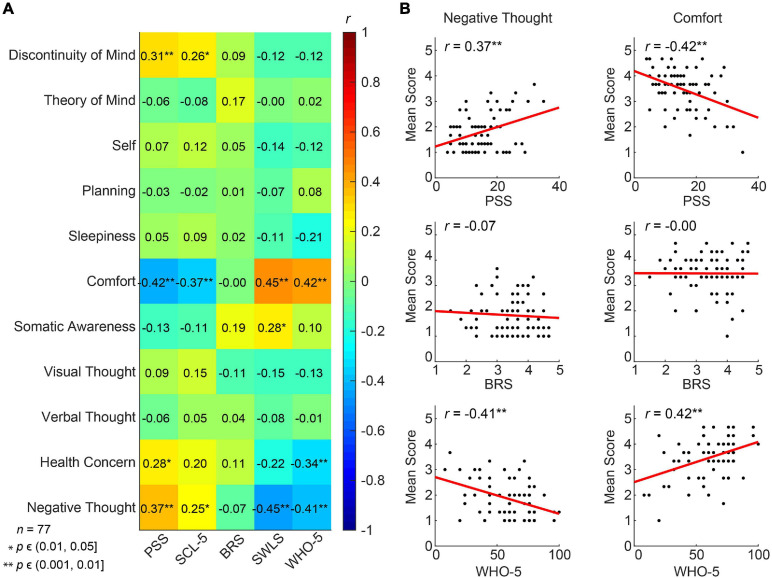
Multiple dimensions of resting-state thoughts and feelings correlate with classical measures of mental well-being. **(A)** Correlation heat map between the 11 ARSQ-derived factors of resting-state cognition and five established psychometric scales related to mental health (Pearson correlation coefficients). Note the strong and opposite correlations between the factors Negative Thought and Comfort and the classical scales. **(B)** Comfort and Negative Thought show opposite correlations with the WHO-5 scale probing mental well-being in the past two weeks, where high scores should be interpreted as good mental health. Similarly, Comfort and Negative Thought show opposite correlations with the PSS, a scale measuring the amount of stress one experienced in the past month. Interestingly, the BRS measure of general resilience shows no correlation with either Negative Thought or Comfort.

We further tested, looking at the baseline results (T0) from the total cohort of 82 pre-retirement employees, whether thoughts and feelings at rest as reflected in the ARSQ are associated with PSS, BRS, WHO-5, SCL-5 and SWLS scores. Resting-state Comfort correlated positively with life satisfaction (SWLS, *r* = 0.45, *p* = 0.01) and mental well-being in recent weeks (WHO-5, *r* = 0.42, *p* = 0.01), whereas correlations with perceived stress and symptoms of emotional distress, anxiety and depression were negative (PSS and SCL-5) (Figure 6). In agreement with these findings, we observed the opposite correlations for Negative Thought—an experimental dimension developed for the present study (Figure 6). Correlations with Discontinuity of Mind and Health Concern also reached significance for the PSS and the latter also for the WHO-5 scale. Interestingly, whereas resilience showed significant improvement after the MBSR intervention, the BRS exhibited no correlation with any of the ARSQ dimensions, perhaps reflecting the circumstance that the items in the BRS denote a certain way of relating to and coping with stressful events, not easily reflected in certain typical thoughts and feelings, such as framed in the ARSQ.

**FIGURE 7:**
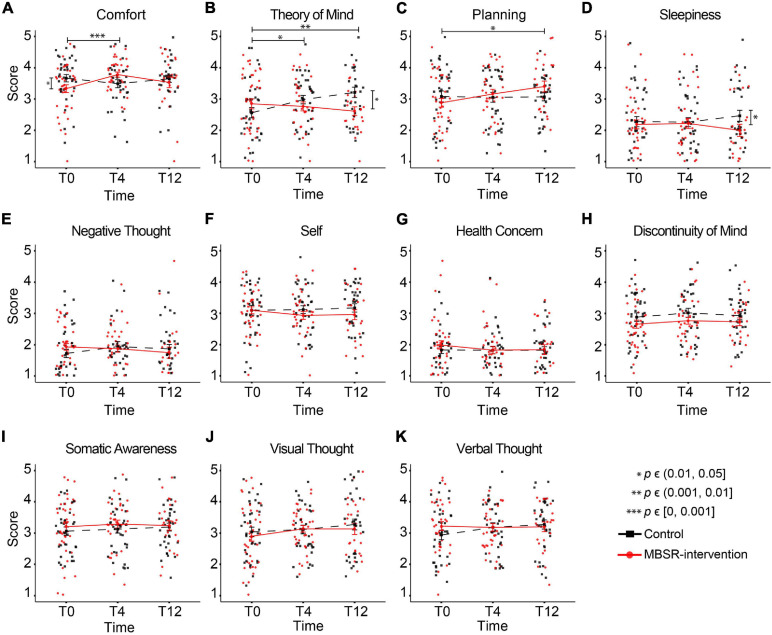
Increasing resting-state Comfort in the MBSR compared to the Control group. Changes in the group-mean responses over time for the eleven ARSQ dimensions **(A–K)** are shown. Significant differences in the estimated changes over time in the two groups are shown with horizontal bars and asterisks. Vertical bars and asterisks indicate a significant difference between the group sampling means at each individual time point. All statistics are based on LMM, see Table 3 for details. T0: baseline measurement; T4 and T12: measurements after 4 and 12 months, respectively.

To test specifically whether the MBSR program would alter patterns of thoughts and feelings that were previously found to be reproducible (Diaz et al., 2013), we performed LMM analysis on the 11 ARSQ dimensions (see Methods and Figure 3). Most notably, Comfort scores for the MBSR group showed a significant increase at T4 compared to Controls (Table 3 and Figure 7A), which is in line with the positive correlations between Comfort and WHO-5 (Figure 6) and the effect of MBSR according to the WHO-5 scale. We also note that participants scoring low on Comfort at baseline (T0) seemed to benefit the most with the number of participants (scoring below 3 on Comfort) decreasing from 11 to 3. LMM also revealed significant difference in Theory of Mind in the MBSR group compared to Controls at time T4 (mean change difference −0.49, 95% CI = [−0.97, −0.01], *p* = 0.049) and T12 (mean change difference 0.82, 95% CI = [−1.34, −0.3], *p* = 0.003, Table 3 and Figure 7B), and a significant increase in Planning for the MBSR group compared to Controls at T12 (mean change difference 0.6, 95% CI = [0.08, 1.11], *p* = 0.025, Table 3 and Figure 7C). We did not observe any mean change differences between the two groups for the other eight ARSQ dimensions (Figures 7D–K). For a complete report of the LMM analysis of all ARSQ dimensions, see Supplementary Table 4.

**TABLE 3:** The MBSR-intervention group shows increased Comfort at T4 compared to the Control group.

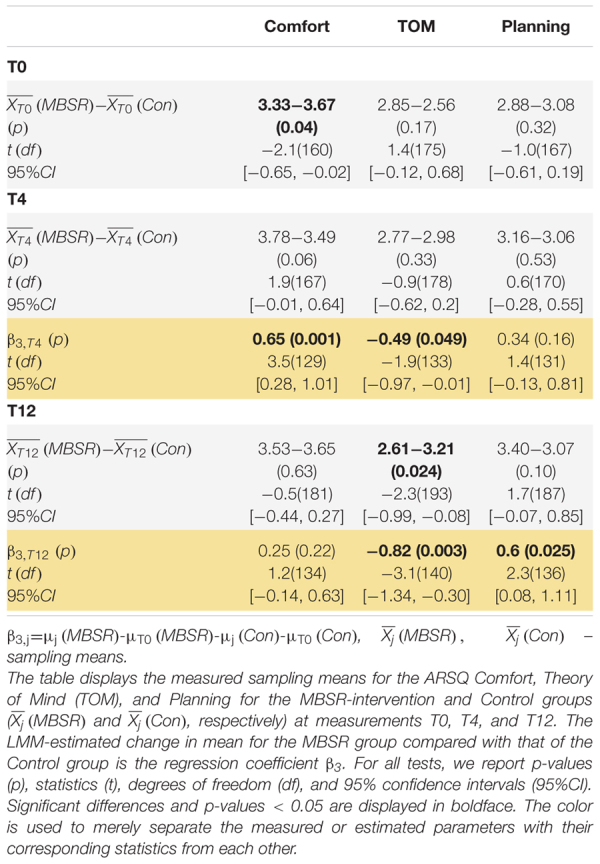

## Discussion

In the present study, we used five validated self-report questionnaires to investigate the effect of MBSR on perceived stress, resilience, symptoms of emotional distress, anxiety and depression, current well-being, and satisfaction with life in healthy 60–65-year-old pre-retirement employees, compared to a waitlist control group. We build upon and extend the findings on the role of mindfulness interventions in the workplace and argue that there are many benefits that are derived from cost-effective MBSR intervention for pre-retirement employees. In accordance with our prediction, MBSR participants experienced improved resilience and mental well-being, compared to the waitlist, not only at the end of the intervention (after 4 months), but also at the 12-month follow-up measurement. Futhermore, MBSR participants also showed trends of improvements on perceived stress, symptoms of emotional distress, anxiety and depression, and satisfaction with life. These psychometric variables showed meaningful baseline associations with responses on the ARSQ, which we used to sample thoughts and feelings during a 5-minute eyes-closed rest session. In agreement with our second prediction, the MBSR group increased significantly resting-state Comfort and had improvements in sleepiness. However, MBSR training did not induce decreases in Discontinuity of Mind and Negative Thought which may be due to our cohort not reporting high levels of Discontinuity of Mind or Negative Thought at baseline, which left little room for improvement.

### Healthy Pre-retirement Employees Are in Need of Stress Reduction

The participants had an active work life at the time of enrollment. While this status could imply a high level of resourcefulness and no great need for improvement, the questionnaire scores told a different story with 46% of participants experiencing moderate stress and 10% experiencing high stress. Norm scores based on an American population show that PSS scores generally decrease with one point per decade to reach its lowest (11.9) at 55–64 years of age, followed by a rise from age 65+ (Cohen, 1988). Hence, although the mean PSS score (15.7) was well below 18, which has been associated with higher mortality within a four-year period (Prior et al., 2016), our group of participants were on the high end of the expected stress scores for their age. In agreement with the fairly high level of perceived stress, 25% of participants experienced poor well-being (23% of participants showed both moderate to high level of stress and poor well-being). The scales measuring resilience, satisfaction with life, or symptoms of anxiety and depression also identified approximately a quarter of participants to have critically poor scores. Hence, the baseline measures indicated that our group of participants was actually a relevant group with a potential for improvement. This could be explained by self-selection effects, as participants signed up for the study voluntarily. Through the questionnaires, participants—whether they were aware of this or not—revealed a need for intervention.

### MBSR Training Leads to Lasting Improvements in Resilience and Well-Being

After MBSR training, the participants reported lower perceived stress levels than Controls both at T4 and T12; however, the difference of the magnitude of change between the two groups did not reach significance in the LMM analysis. Hence, following the MBSR training, we did not observe a superior change in perceived stress, the primary outcome variable of this study. This lack of significant differences between groups in the changes over time most likely resulted from the lack of power due to the reduced sample size, which was a consequence of COVID-19 restrictions (see Figure 2).

The MBSR intervention also did not induce significant differences between the groups in the changes over time in symptoms of psychological distress, and symptoms of anxiety and depression (SCL-5). At T0, the average scores were lingering below the SCL-5 cut-point (2) above which mental illness can be predicted (Strand et al., 2003). However, the SCL-5 took an upward course in the control group while decreasing steadily in the MBSR group, to reach a significant sample mean difference at T12, which could tentatively be interpreted suggest the workings of a more time-demanding consolidation process.

Importantly, we observed significant and lasting improvements for WHO-5 and BRS. The control group showed trends of worsening symptoms measured by PSS and SCL-5, whereas the MBSR group stayed relatively stable. This may be due to the protection mechanisms offered by the intervention which is shown by the increase of well-being and resilience of the MBSR group. The MBSR group improved their scores of well-being and resilience especially at T4 and stayed significant at T12. Resilience has been demonstrated to have protective capacities that are related to better mental health in the future (Nila et al., 2016), and there is an increasing focus on improvements in resilience with regards to protecting the long-term well-being of employees (Harvey et al., 2014). This perspective, while being relevant to all age groups, carries special relevance to aging employees who are exposed to additional age-related stress and associated lowered resilience to illnesses. MBSR hence offers aging employees a means to maintain a healthy work-life in the last years before retirement.

Several studies have linked the COVID-19 pandemic to increasing psychological distress in the general population, escalating symptoms of depression, anxiety, and stress (Ettman et al., 2020; Xiong et al., 2020). Although the COVID-19 may have decreased the external validity of our results, it is a tempting interpretation that the special extra stressful circumstances induced by COVID-19 may specifically have worked against improvements on the PSS, which emphasize lack of control. However, notwithstanding COVID-19, the control group did not show a significant change of stress levels from baseline, suggesting that the pandemic probably did not act as an aggravating factor. Taking the results at face value, it becomes clear that MBSR participants report selective improvements in stress coping in the sense of being able to not let stressful events get to them, i.e., letting the events pass and move on, another word for this is *resilience*. The MBSR program has consistently been shown to decrease self-reported distress and stress post-intervention (Khoury et al., 2015), perhaps reflecting the predominant use of questionnaires that sample stressful experiences. However, especially noteworthy in the present study is the finding that resilience increases and stays high, while the control group remains unchanged. This suggests that the BRS scale captures a central beneficial outcome of the MBSR program. The items in the BRS collectively emphasize the tendency to bounce back quickly after hard times, and to recover quickly from stressful events with little trouble. The mechanisms involved in the achieved boost in resilience may count, in particular, training of acceptance skills in MBSR, i.e., acknowledging the habitual reactions to stressful situations with a non-judgmental, matter-of-fact attitude, eventually discovering that mindful awareness allows for additional choices in response to stress (Lindsay and Creswell, 2017; Chin et al., 2019). The implied re-perception has been associated with improved self-regulation, clarification of values, and cognitive, emotional, and behavioral flexibility (Shapiro et al., 2006).

### MBSR Participants With Previous Yoga/Meditation Experience Drive the Main Effect of the MBSR Intervention

On comparison of the experienced with inexperienced participants (i.e., participants with and without previous yoga/meditation experience at the beginning of the study, respectively) in the MBSR group, we observed greater improvements in the experienced participants after the MBSR intervention. This, however, could be a result of the significantly poorer baseline BRS, SCL, SWLS, and WHO-5 scores of the experienced MBSR participants as compared to those of the inexperienced MBSR participants. Consequently, the experienced individuals had simply a greater margin to improve their scores from baseline. We speculate that the poorer baseline scores of the experienced MBSR participants reflect that this group may have been seeking yoga/meditation training previously to counter a wide range of mental of physical problems not measured in the present project and recommend a more thorough screening of such a bias in future research. While the improvement in the wellbeing scales of the experienced participants to some extent may be explained by the lack of a “ceiling effect” (i.e., the margin to improve was larger), it is also conceivable that this group with its prior experience with yoga or meditation was more open to the therapy or better prepared to learn the exercises and, therefore, gained more than the inexperienced participants.

### MBSR May Improve Sleep Quality

Since sleep quality is a hallmark symptom of mental health and moderate sleep disturbances in older adults are often associated with deficits in daytime function (Prinz, 1995; McCrae et al., 2005; Ancoli-Israel and Ayalon, 2006), we probed potential effects of MBSR training on sleep. Several studies previously studied the effects of mindfulness on sleep with mixed findings (Winbush et al., 2007; Gross et al., 2011; Hubbling et al., 2014). Our observations showed that the MBSR group had lower sample mean difference in Sleepiness (ARSQ dimension) at T12, and participants’ ratings of experienced sleep quality showed significant improvement in mean change difference at T4. Our results are aligned with a recent systematic review and meta-analysis of randomized controlled trials suggesting that mindfulness meditation may indeed be effective in addressing sleep problems (Rusch et al., 2019). We suggest that future studies should include more extensive sleep scales to further probe the effects of MBSR on sleep quality, in particular in this age group.

### Does General Well-Being Drive Our Thoughts and Feelings in Every Moment?

Using the full data set at baseline (T0), we observed significant correlations between the dimensions Discontinuity of Mind, Comfort, Health Concern, and Negative Thought and the mental health scales PSS, SCL-5, SWLS, and WHO-5. This is not a trivial finding considering that these classical measures examine weeks to months of time, whereas the ARSQ data refer to thoughts and feelings in a 5-minute eye-closed rest period, suggesting that general mental health impacts our feeling of comfort and capacity to control the flow of our thoughts *at any given moment*. This is in line with previous studies analyzing associations between the ARSQ dimensions and mental-health scales. Especially low Comfort and a high degree of Sleepiness or Discontinuity of Mind are characteristic of poor mental health as reflected in clinical psychometric scales measuring anxiety, depression, or sleep quality (Diaz et al., 2013, 2014). Thus, it is plausible that at a multitude of biological mechanisms governing mental health bias our thoughts and feelings at any given moment. This raises the intriguing idea that therapeutic interventions targeting such mechanisms impact momentary patterns of what we feel and think. We indeed observed a highly significant increase in Comfort in the MBSR group compared to Controls at T4. This is an important outcome of the intervention because comfort is essential to mental well-being. One must feel comfortable to maintain focus of attention, including to own thoughts as reflected in the strong negative correlations between Discontinuity of Mind and Comfort (Diaz et al., 2013). Several studies using the ARSQ have reported reduced Comfort in clinical cohorts, including insomnia (Palagini et al., 2016), autism (Simpraga et al., 2021), and also health anxiety and obsessive compulsive disorder (Gehrt et al., 2020). Thus, there is a need and our results indicate a potential for MBSR to facilitate a greater feeling of comfort in these disorders.

The MBSR training did not induce decreases in Discontinuity of Mind, which might be related to a floor effect: our cohort did not report high levels of Discontinuity of Mind at baseline, albeit we know little about the norm values of the ARSQ dimensions in this age group. Similarly, Health Concern and Negative Thought were scored low, which left little room for improvement (Figures 7E,G). We found a significant effect on the dimension Theory of Mind which decreased in the MBSR group, while increasing in the Control group. Theory of Mind is related to the cognitive aspect of empathy, explained as the ability to infer and reflect upon the mental states that underlie other people’s actions (Baron-Cohen, 2000). Theoretic and intervention-based accounts suggest that mindfulness cultivates empathy in a broader sense (Shapiro et al., 1998; Andersen, 2005), also involving affective empathy, which refers to sharing other people’s feelings and to feel emotional concern for other people’s emotions or experiences (Davis, 1983; Duan and Hill, 1996). In any case, taken at face value, the present results suggest that mindfulness makes us *less* prone to “think about others”, “put myself in other peoples’ shoes”, and “think about people I like”. It could be speculated that MBSR participants used the 5 minutes rest of the ARSQ paradigm, which form the basis for the present analysis, to practice moving towards experience, simultaneously moving away from thinking, at T4 and T12, while Controls were not yet familiar with this mindfulness technique. Importantly, this tells little about participants behavior outside the “lab”/ARSQ paradigm.

### Booster Sessions Stimulate Continued Practice

It is well-known that home practice during the MBSR course plays a role, significantly influencing the magnitude of the beneficial outcomes that participants report (Carmody and Baer, 2008; Parsons et al., 2017). In our study, to increase the insight into the home practice behavior in self-referred 60–65-year-old work active Danes, we also inquired about this aspect. After the second data collection (T4), participants in the MBSR group kept up a cadence of 4 weekly home exercise sessions. This was reduced to approximately three times per week during the following 8 months (T12), i.e., a relatively modest reduction of 25%. In this same time period (T4 to T12), well-being (WHO-5) dropped, but resilience remained high, and SCL-5 reached significance in sample mean difference at T12.

It has been observed that many MBSR participants find it hard to sustain a practice after the 8-week course has ended (Carmody and Baer, 2008; Parsons et al., 2017). In our study, the new element—monthly booster sessions—may have played a critical role in the positive results observed after 12 months. As it turned out, 29% of the participants took part in all or most sessions, 37% in some, 5% did not follow any booster sessions, and 29% did not respond. Participants showed no clear preferences regarding the session format, although physical meetings were favored over online sessions, and a good third of the participants preferred a mixed setup.

### Limitations

#### Reduced Sample Size and Lack of Long-Term Follow-Up

The current literature examining mindfulness-based interventions for older adults is scarce and lacking randomized controlled studies with sufficiently large sample sizes and long-term follow-ups (Klimecki et al., 2019). The present study was designed to meet these needs, but unfortunately both the sample size and the long-term follow-up were compromised by the COVID-19 pandemic. The sample size of the study was reduced to 82 from originally 192 participants, resulting from a power calculation based on the primary outcome measure PSS with a sensitivity limit set to at least a clinically moderate effect size. Due to the reduced sample size, we did not adjust for multiple comparisons. Still, the observation of moderate improvements in a broad range of effect measures—many of them reaching statistical significance in spite of the loss of statistical power—is promising and makes a strong case for adequately powered follow-up studies. Also due to the reduced sample size, there was also insufficient power to analyze sub-groups.

#### Booster Sessions Were Added ad hoc, and Did Not Require Participation

In the present study, the booster sessions were added ad hoc as an attempt to keep the MBSR practice active until COVID restrictions were halted and follow-up physiological data collection (including MR brain scans) could again take place. Unfortunately, this opportunity did not arrive within an acceptable time frame, and the study stopped a year before planned. The booster sessions, although offering a very interesting addition to the 8-week MBSR program, could not be tested in the optimal manner by making the sessions mandatory to all participants, due to the ethical problem in adding new elements after participants had already signed up to the original protocol. An additional analysis comparing the effects of attendance versus no attendance in the booster sessions could reveal if and to what extent these booster sessions had their own effect. Unfortunately, the present dataset does not offer the statistical power to isolate the effects of the booster sessions because only 2 people from the MBSR group did not participate in any of the booster sessions. In a future study the effect of booster sessions should be part of the original protocol and studied in its own right, relying on full, or close-to-full participation.

#### Multi-Method Approach Was Reduced to Single-Method Questionnaire Approach

The exclusion of physiological data after the initial baseline collection of anthropometric, cardiovascular and morphological and functional brain imaging data limited the present study in a highly regrettable manner, as these measures were included to further our neurobiological understanding of MBSR-induced health benefits in this important but overlooked cohort. Future research should aim to address the aforementioned shortcomings with the inclusion of physiological and morphological or functional brain imaging data that had to be abandoned in the present study due to COVID-19.

#### Outlook

In spite of the adverse impact of the COVID-19 pandemic on our randomized control trial, we observed promising effects of MBSR on well-being and resilience of 60–65-year-old employees in the private sector. The pandemic stimulated the development of a new booster-session MBSR format, which may have contributed to the lasting improvements on resilience and well-being at the 12-months follow-up. The participants were characterized by high job satisfaction and generally had few sick days from work; however, at least a quarter of the participants scored in a critical range on mental health scales. They gladly signed up for a free 8-week MBSR program and the majority also showed up for monthly booster sessions. Thus, the booster-session format should be considered in the future, not only in scientific studies but also to sustain older working people’s engagement, continued practice, and improved health status, which is an important challenge (Masheder et al., 2020). We recommend larger and longer-running studies to investigate whether the health benefits of MBSR would affect how long older employees stay in their work-life. In addition to the overall greater statistical power, a larger sample would also allow for subgroup analyses, such as baseline stratification investigating the profiles of the participants who benefit the most from the MBSR intervention. From a practical and economic point of view—not the least for employers—we noted in our data a considerable potential in this respect.

## Data Availability Statement

The raw data supporting the conclusions of this article will be made available by the authors, without undue reservation.

## Ethics Statement

The studies involving human participants were reviewed and approved by Danish Research Ethics Committee. The patients/participants provided their written informed consent to participate in this study.

## Author Contributions

KJP and NH conceived and designed the study and organized and collected the data. KS and MD performed the statistical analysis and created the figures and tables. NH and LF organized the Mindfulness-Based Stress Reduction (MBSR) intervention. KJP, KL-H, KS, MD, and NH wrote sections of the manuscript. KJP, KL-H, KS, MD, LF and NH contributed to manuscript revision, read and approved the submitted version. All authors contributed to the article and approved the submitted version.

## Conflict of Interest

The authors declare that the research was conducted in the absence of any commercial or financial relationships that could be construed as a potential conflict of interest.
