# Supplementary material for: Pre-retirement Employees Experience Lasting Improvements in Resilience and Well-Being After Mindfulness-Based Stress Reduction
Source: Front Psychol. 2021 Jul 15;12:699088. doi: 10.3389/fpsyg.2021.699088 (PMC8321239; doi:10.3389/fpsyg.2021.699088)
Supplement: Supplementary file 1 [file Table_1.docx]

***Supplementary Material***

# Supplementary Table

**Supplementary Table S1. The MBSR-intervention group shows superior and lasting improvements in resilience and well-being compared to the Control group.** The table displays the measured sampling means for each of the five psychometric scales for the MBSR-intervention and Control groups ($\bar{X_{j}}\left( MBSR \right)$ and $\bar{X_{j}}\left( Con \right)$, respectively) at measurements T0, T4, and T12. The LMM-estimated mean for the Control group at T0 is the regression coefficient $\beta_{0}$, and the estimated mean difference between the MBSR and Control group is the regression coefficient $\beta_{1}$. Regression coefficient $\beta_{2}$ is the estimated change in mean for the Control group. The LMM-estimated change in mean for the MBSR group compared with that of the Control group is the regression coefficient $\beta_{3}$. For all tests, we report p-values (*p*), statistics (*t*), degrees of freedom (*df*), and 95% confidence intervals (95%*CI)*. Significant differences and p-values < 0.05 are displayed in boldface. The last block (T12–T4) shows sampling and estimated differences in means between the follow-up at T12 and post-intervention at T4 for either group. Significant differences and p-values < 0.05 are displayed in boldface. The color is used to merely separate the measured or estimated parameters with their corresponding statistics from each other.

|  | PSS | BRS | WHO-5 | SCL-5 | SWLS |
| --- | --- | --- | --- | --- | --- |
| T0 |  |  |  |  |  |
| $\bar{X_{T0}}\left( MBSR \right)$-$\bar{X_{T0}}\left( Con \right)$  $(p)$  $t\left( df \right)$  $95\%CI$ | 14.72-16.05  (0.37)  -0.9(136)  [-4.02, 1.49] | 3.55-3.45  (0.53)  0.6(113)  [-0.21, 0.42] | 62.40-62.05 (0.93)  0.1(144)  [-7.94, 8.67] | 1.8-1.88  (0.49)  -0.7(154)  [-0.31, 0.15] | 26.08-24.59 (0.26)  1.1(113)  [-1.12, 4.16] |
| $\beta_{0} (p)$  $t\left( df \right)$  $95\%CI$ | 15.99 (<.001)  16.0(137),  [14.05, 17.93] | 3.45 (<.001)  30.2(113)  [3.23, 3.67] | 62.05 (<.001)  20.7(143)  [56.20, 67.90] | 1.88 (<.001)  22.4(154)  [1.72, 2.04] | 24.59 (<.001)  25.7(112)  [22.72, 26.45] |
| $\beta_{1} (p)$  $t\left( df \right)$  $95\%CI$ | -1.27 (0.37)  -0.9(136)  [-4.02, 1.49] | 0.1 (0.53)  0.6(113)  [-0.21, 0.42] | 0.36 (0.93)  0.1(144)  [-7.94, 8.67] | -0.08 (0.49)  -0.7(154)  [-0.31, 0.15] | 1.52 (0.26)  1.1(113)  [-1.12, 4.16] |
| T4 |  |  |  |  |  |
| $\bar{X_{T4}}\left( MBSR \right)$-$\bar{X_{T4}}\left( Con \right)$  $(p)$  $t\left( df \right)$  $95\%CI$ | **12.92-16.53 (0.026)**  -2.2(139)  [-6, -0.42] | **3.87-3.34**  **(0.003)**  3.1(119)  [0.18, 0.83] | **71.47-59.80 (0.010)**  2.6(148)  [2.84, 19.66] | 1.56-1.72  (0.20)  -1.3(160)  [-0.39, 0.08] | 27.45-25.13 (0.11)  1.6(115)  [-0.49, 4.84] |
| $\beta_{2,T4} (p)$  $t\left( df \right)$  $95\%CI$ | 0.22 (0.81)  0.2(137)  [-1.61, 2.06] | -0.09 (0.31)  -1.0(134)  [-0.26, 0.08] | -2.00 (0.49)  -0.7(138)  [-7.61, 3.6]; | -0.16 (0.07)  -1.8(139)  [-0.33, 0.01] | 0.66 (0.35)  0.9(136)  [-0.7, 2.01] |
| $\beta_{3,T4} (p)$  $t\left( df \right)$  $95\%CI$ | -1.95 (0.14)  -1.5(136)  [-4.52, 0.62] | **0.4 (0.002)**  3.2(135)  [0.16, 0.65] | **10.90 (0.009)**  2.6(140)  [2.86, 18.95] | -0.07 (0.56)  -0.6(139)  [-0.31, 0.17] | 0.66 (0.51)  0.7(137)  [-1.29, 2.60] |
| T12 |  |  |  |  |  |
| $\bar{X_{T12}}\left( MBSR \right)$-$\bar{X_{T12}}\left( Con \right)$  $(p)$  $t\left( df \right)$  $95\%CI$ | **12.00-15.26 (0.049)**  -1.9(165)  [-6.08, -0.05] | **3.90-3.44**  **(0.033)**  2.2(140)  [0.04, 0.71] | **64.80-56.33 (0.032)**  2.2(176)  [1.06, 19.48] | **1.48-1.77**  **(0.027)**  -2.2(184)  [-0.55, -0.04] | 26.15-24.20 (0.14)  1.5(136)  [-0.69, 4.94] |
| $\beta_{2,T12} (p)$  $t\left( df \right)$  $95\%CI$ | -0.79 (0.42)  -0.8(139)  [-2.67, 1.1] | 0.01 (0.89) 0.1(135)  [-0.16, 0.19] | -5.84 (0.05)  -1.9(140)  [-11.65, -0.03] | -0.1 (0.28)  -1.1(141)  [-0.27, 0.08] | -0.01 (0.99)  -0.01(137)  [-1.43, 1.41] |
| $\beta_{3,T12} (p)$  $t\left( df \right)$  $95\%CI$ | -1.80 (0.22)  -1.5(140)  [-4.62, 1.01] | **0.27 (0.049)** 3.2(137)  [0.01, 0.54] | **9.89 (0.031)**  2.6(143)  [1.02, 18.74] | -0.21 (0.12)  -1.6(144)  [-0.48, 0.05] | 0.60 (0.59)  0.7(139)  [-1.54, 2.75] |
| T12-T4 |  |  |  |  |  |
| $\bar{X_{T12}}\left( Con \right)$-$\bar{X_{T4}}\left( Con \right)$ | 15.26-16.53 | 3.44-3.34 | 56.33-59.80 | 1.77-1.72 | 24.20-25.12 |
| $\bar{X_{T12}}\left( MBSR \right)$-$\bar{X_{T4}}\left( MBSR \right)$ | 12.00-12.92 | 3.9-3.87 | 64.8-71.47 | 1.48-1.56 | 26.15-27.45 |
| $\Delta_{con} (p)$  $t\left( df \right)$  $95\%CI$ | -1.01 (0.30)  -1.0(136)  [-2.91, 0.89] | 0.10 (0.27)  1.1(134)  [-0.08, 0.28] | -3.84 (0.20)  -1.3(139)  [-9.66, 2.00] | 0.06 (0.51)  0.7(138)  [-0.12, 0.24] | -0.67 (0.36)  -0.9(137)  [-2.09, 0.76] |
| $\Delta_{MBSR} (p)$  $t\left( df \right)$  $95\%CI$ | -0.86 (0.43)  -0.8(140)  [-2.96, 1.23] | -0.03 (0.79)  -0.3(138)  [-0.23, 0.18] | -4.84 (0.17)  -1.4(146)  [-11.61, 1.90] | -0.08 (0.43)  -0.8(146)  [-0.28, 0.12] | -0.72 (0.39)  -0.9(140)  [-2.34, 0.90] |

$\beta_{0}=\mu_{T0}\left( Con \right)$, $\beta_{1}=\mu_{T0}\left( MBSR \right)$-$\mu_{T0}\left( Con \right)$, $\beta_{2,j}=\mu_{j}\left( Con \right)$-$\mu_{T0}\left( Con \right)$,

$\beta_{3,j}=\mu_{j}\left( MBSR \right)$-$\mu_{T0}\left( MBSR \right)$-$\mu_{j}\left( Con \right)$-$\mu_{T0}\left( Con \right)$,

$\Delta_{Con}=\mu_{T12}\left( Con \right)$-$\mu_{T4}\left( Con \right)$, $\Delta_{MBSR}=\mu_{T12}\left( MBSR \right)$-$\mu_{T4}\left( MBSR \right)$,

$\bar{X_{j}}\left( MBSR \right), \bar{X_{j}}\left( Con \right)$ – sampling means
