# Supplementary material for: Pre-retirement Employees Experience Lasting Improvements in Resilience and Well-Being After Mindfulness-Based Stress Reduction
Source: Front Psychol. 2021 Jul 15;12:699088. doi: 10.3389/fpsyg.2021.699088 (PMC8321239; doi:10.3389/fpsyg.2021.699088)
Supplement: Supplementary file 2 [file Table_2.docx]

***Supplementary Material***

# Supplementary Table

**Supplementary Table S2. The MBSR-intervention participants with previous yoga/meditation experience show significantly poorer baseline BRS, SCL-5, SWLS, and WHO-5 scores as compared to MBSR-intervention participants without previous practice and consequently gain more improvement on these psychometric outcomes after the intervention.** The table displays the measured sampling means for each of the five psychometric scales for experienced participants and inexperienced participants in the MBSR group ($\bar{X_{j}}\left( MBSR/Exp \right)$ and $\bar{X_{j}}\left( MBSR/Inexp \right)$, respectively) at measurements T0, T4, and T12. The LMM-estimated mean for the MBSR group of participants with previous experience at T0 is the regression coefficient $\beta_{0}$, and the estimated mean difference between the MBSR-intervention individuals without experience and MBSR-intervention individuals with experience is the regression coefficient $\beta_{1}$. Regression coefficient $\beta_{2}$ is the estimated change in mean for the experienced participants of the MBSR group. The LMM-estimated change in mean for the inexperienced participants of the MBSR group compared with that of the experienced individuals is the regression coefficient $\beta_{3}$. For all tests, we report p-values (*p*), statistics (*t*), degrees of freedom (*df*), and 95% confidence intervals (95%*CI)*. The last block (T12–T4) shows sampling and estimated differences in means between the follow-up at T12 and post-intervention at T4 for either category of participants. Significant differences and p-values < 0.05 are displayed in boldface. The color is used to separate the measured or estimated parameters with their corresponding statistics from each other.

|  | PSS | BRS | SCL-5 | SWLS | WHO-5 |
| --- | --- | --- | --- | --- | --- |
| T0 |  |  |  |  |  |
| $\bar{X_{T0}}\left( MBSR/Inexp \right)$-$\bar{X_{T0}}\left( MBSR/Exp \right)$  $(p)$  $t\left( df \right)$  $95\%CI$ | 13.67-15.36  (0.391)  -0.86(63)  [-5.49, 2.1] | 4.01-3.26  **(<.001)**  3.93(67)  [0.38, 1.12] | 1.59-1.93  **(0.035)**  -2.14(73)  [-0.65, -0.03] | 29.62-23.71  **(0.001)**  3.44(62)  [2.55, 9.12] | 72.00-56.00  **(0.007)**  2.78(71)  [4.89, 27.05] |
| $\beta_{0} (p)$  $t\left( df \right)$  $95\%CI$ | 15.36 (<.001)  12.79(63)  [13.04, 17.68] | 3.26 (<.001)  27.3(67)  [3.03, 3.49] | 1.93 (<.001)  19.42(73)  [1.74, 2.12] | 23.79 (<.001)  22.33(62)  [21.73, 25.85] | 56.03 (<0.001)  15.5(71)  [49.05, 63.01] |
| $\beta_{1} (p)$  $t\left( df \right)$  $95\%CI$ | -1.69 (0.391)  -0.86(63)  [-5.49, 2.1] | **0.75 (<.001)**  3.93(67)  [0.38, 1.12] | **-0.34 (0.035)**  -2.14(73)  [-0.65, -0.03] | **5.84 (0.001)**  3.44(62)  [2.55, 9.12] | **15.97 (0.007)**  2.78(71)  [4.89, 27.05] |
| T4 |  |  |  |  |  |
| $\bar{X_{T4}}\left( MBSR/Inexp \right)$-$\bar{X_{T4}}\left( MBSR/Exp \right)$  $(p)$  $t\left( df \right)$  $95\%CI$ | 12.2-13.38  (0.514)  -0.66(64)  [-5.11, 2.52] | **4.2-3.67**  **(0.007)**  2.76(72)  [0.17, 0.93] | 1.49-1.61  (0.512)  -0.66(77)  [-0.42, 0.21] | 29.27-26.26  (0.087)  1.74(63)  [-0.34, 6.32] | 72.27-70.96  (0.789)  0.27(72)  [-9.71, 12.86] |
| $\beta_{2,T4} (p)$  $t\left( df \right)$  $95\%CI$ | -1.87 (0.09)  -1.72(63)  [-3.97, 0.22] | **0.39 (0.002)**  3.29(60)  [0.16, 0.62] | **-0.32 (0.003)**  -3.09(64)  [-0.52, -0.12] | **2.46 (0.01)**  2.67(61)  [0.69, 4.24] | **14.67 (<.001)**  4.08(63)  [7.75, 21.59] |
| $\beta_{3,T4} (p)$  $t\left( df \right)$  $95\%CI$ | 0.4 (0.82)  0.23(63)  [-2.98, 3.79] | -0.2 (0.303)  -1.04(60)  [-0.57, 0.17] | 0.23 (0.164)  1.41(64)  [-0.09, 0.55] | -2.85 (0.055)  -1.95(61)  [-5.65, -0.04] | **-14.4 (0.014)**  -2.54(63)  [-25.34, -3.47] |
| T12 |  |  |  |  |  |
| $\bar{X_{T12}}\left( MBSR/Inexp \right)$-$\bar{X_{T12}}\left( MBSR/Exp \right)$  $(p)$  $t\left( df \right)$  $95\%CI$ | 11.36-12.47  (0.693)  -0.4(81)  [-5.12, 3.37] | 4-3.83  (0.219)  1.24(88)  [-0.15, 0.72] | 1.51-1.47  (0.632)  0.48(91)  [-0.27, 0.45] | 27.27-25.33  (0.309)  1.02(78)  [-1.72, 5.61] | 64.8-64.8  (0.863)  -0.17(89)  [-14.15, 11.79] |
| $\beta_{2,T12} (p)$  $t\left( df \right)$  $95\%CI$ | **-2.9 (0.028)**  -2.24(66)  [-5.39, -0.41] | **0.48 (0.001)**  3.44(63)  [0.21, 0.75] | **-0.49 (<.001)**  -4.01(67)  [-0.72, -0.25] | 2.17 (0.05)  2(64)  [0.07, 4.25] | **10.87 (0.011)**  2.61(65)  [2.82, 18.85] |
| $\beta_{3,T12} (p)$  $t\left( df \right)$  $95\%CI$ | 0.82 (0.685)  0.41(65)  [-3.06, 4.7] | **-0.47 (0.038)**  -2.12(63)  [-0.9, -0.04] | **0.43 (0.025)**  2.29(67)  [0.07, 0.79] | **-3.89 (0.023)**  -2.34(63)  [-7.1, -0.68] | **-17.14 (0.011)**  -2.61(65)  [-29.77, -4.46] |
| T12-T4 |  |  |  |  |  |
| $\bar{X_{T12}}\left( MBSR/Exp \right)$-$\bar{X_{T4}}\left( MBSR/Exp \right)$ | 12.47-13.38 | 3.83-3.67 | 1.47-1.61 | 25.33-26.26 | 64.8-70.96 |
| $\bar{X_{T12}}\left( MBSR/Inexp \right)$-$\bar{X_{T4}}\left( MBSR/Inexp \right)$ | 11.36-12.20 | 4.00-4.20 | 1.51-1.49 | 27.27-29.27 | 64.8-72.27 |
| $\Delta_{MBSR/Exp} (p)$  $t\left( df \right)$  $95\%CI$ | -1.03 (0.429)  -0.8(65)  [-3.53, 1.47] | 0.09 (0.535)  0.62(63)  [-0.18, 0.36] | -0.17 (0.184)  -1.34(68)  [-0.4, 0.07] | -0.29 (0.789)  -0.27(63)  [-2.39, 1.8] | -3.8 (0.371)  -0.9(66)  [-11.98, 4.3] |
| $\Delta_{MBSR/Inexp} (p)$  $t\left( df \right)$  $95\%CI$ | -0.61 (0.693)  -0.4(64)  [-3.59, 2.36] | -0.18 (0.305)  -1.03(61)  [-0.52, 0.16] | 0.03 (0.828)  0.22(65)  [-0.25, 0.31] | -1.34 (0.296)  -1.05(62)  [-3.8, 1.1] | -6.54 (0.206)  -1.28(64)  [-16.41, 3.31] |

$\beta_{0}=\mu_{T0}\left( MBSR/Exp \right)$, $\beta_{1}=\mu_{T0}\left( MBSR/Inexp \right)$-$\mu_{T0}\left( MBSR/Exp \right)$, $\beta_{2,j}=\mu_{j}\left( MBSR\boldsymbol{/}Exp \right)$-$\mu_{T0}\left( MBSR\boldsymbol{/}Exp \right)$,

$\beta_{3,j}=\mu_{j}\left( MBSR/Inexp \right)$-$\mu_{T0}\left( MBSR/Inexp \right)$-$\mu_{j}\left( MBSR\boldsymbol{/}Exp \right)$-$\mu_{T0}\left( MBSR\boldsymbol{/}Exp \right)$,

$\Delta_{MBSR\boldsymbol{/}Exp}=\mu_{T12}\left( MBSR\boldsymbol{/}Exp \right)$-$\mu_{T4}\left( MBSR\boldsymbol{/}Exp \right)$, $\Delta_{MBSR/Inexp}=\mu_{T12}\left( MBSR/Inexp \right)$-$\mu_{T4}\left( MBSR/Inexp \right)$,

$\bar{X_{j}}\left( MBSR/Inexp \right), \bar{X_{j}}\left( MBSR\boldsymbol{/}Exp \right)$ – sampling means
