# Supplementary material for: Pre-retirement Employees Experience Lasting Improvements in Resilience and Well-Being After Mindfulness-Based Stress Reduction
Source: Front Psychol. 2021 Jul 15;12:699088. doi: 10.3389/fpsyg.2021.699088 (PMC8321239; doi:10.3389/fpsyg.2021.699088)
Supplement: Supplementary file 3 [file Table_3.docx]

***Supplementary Material***

# Supplementary Table

# Supplementary Table S3. Linear mixed model analyses show significant sleep improvement for MBSR group and an overall high job satisfaction. The table displays the measured sampling means for the Sleep quality and Job Satisfaction for the MBSR-intervention and Control groups ($\bar{X}_{j}\left( MBSR \right)$ and $\bar{X}_{j}\left( Con \right)$, respectively) at measurements T0, T4, and T12. The LMM-estimated mean for the Control group at T0 is the regression coefficient $\beta_{0}$, and the estimated mean difference between the MBSR and Control group is the regression coefficient $\beta_{1}$. Regression coefficient $\beta_{2}$ is the estimated change in mean for the Control group. The LMM-estimated change in mean for the MBSR group compared with that of the Control group is the regression coefficient $\beta_{3}$. For all tests, we report p-values (*p*), statistics (*t*), degrees of freedom (*df*), and 95% confidence intervals (95%*CI)*. The last block (T12–T4) shows sampling and estimated differences in means between the follow-up at T12 and post-intervention at T4 for either group. Significant differences and p-values < 0.05 are displayed in boldface. The color is used to merely separate the measured or estimated parameters with their corresponding statistics from each other.

|  | Sleep Quality | Job satisfaction |
| --- | --- | --- |
| T0 |  |  |

| $\bar{X_{T0}}\left( MBSR \right)$*-*$\bar{X_{T0}}\left( Con \right)$  $(p)$  $t\left( df \right)$  $95\%CI$ | 3.32-3.44  (0.47)  -0.7(132)  [-0.45, 0.2] | 3.95-4  (0.84)  -0.2(193)  [-0.53, 0.43] |
| --- | --- | --- |
| $\beta_{0} (p)$  $t\left( df \right)$  $95\%CI$ | 3.44 (<0.001)  29.2(132)  [3.21, 3.67] | 4 (<0.001)  22.9(193)  [3.66, 4.34] |
| $\beta_{1} (p)$  $t\left( df \right)$  $95\%CI$ | -0.12 (0.47)  -0.7(132)  [-0.45, 0.2] | -0.05 (0.84)  -0.2(193)  [-0.53, 0.43] |
| T4 |  |  |
| $\bar{X_{T4}}\left( MBSR \right)$-$\bar{X_{T4}}\left( Con \right)$  $(p)$  $t\left( df \right)$  $95\%CI$ | 3.71-3.51  (0.24)  1.2(132)  [-0.13, 0.52] | 3.77-3.44  (0.16)  1.4(194)  [-0.13, 0.83] |
| $\beta_{2,T4} (p)$  $t\left( df \right)$  $95\%CI$ | 0.07 (0.48)  0.7(147)  [-0.13, 0.27] | **-0.56 (0.007)**  -2.7(147)  [-0.96, -0.16] |
| $\beta_{3,T4} (p)$  $t\left( df \right)$  $95\%CI$ | **0.32 (0.03)**  2.2(147)  [0.04, 0.6] | 0.4 (0.18)  1.4(147)  [-0.17, 0.97] |
| T12 |  |  |
| $\bar{X_{T12}}\left( MBSR \right)$-$\bar{X_{T12}}\left( Con \right)$  $(p)$  $t\left( df \right)$  $95\%CI$ | 3.61-3.62  (0.64)  0.5(152)  [-0.26, 0.42] | 3.58-3.78  (0.64)  -0.5(208)  [-0.65, 0.4] |
| $\beta_{2,T12} (p)$  $t\left( df \right)$  $95\%CI$ | 0.16 (0.12)  1.6(148)  [-0.04, 0.37] | -0.23 (0.29)  -1.1(150)  [-0.64, 0.19] |
| $\beta_{3,T12} (p)$  $t\left( df \right)$  $95\%CI$ | 0.2 (0.19)  1.3(150)  [-0.1, 0.5] | -0.08 (0.8)  -0.3(153)  [-0.69, 0.52] |
| T12-T4 |  |  |
| $\bar{X_{T12}}\left( Con \right)$-$\bar{X_{T4}}\left( Con \right)$ | 3.62-3.51 | 3.78-3.44 |
| $\bar{X_{T12}}\left( MBSR \right)$-$\bar{X_{T4}}\left( MBSR \right)$ | 3.61-3.71 | 3.58-3.77 |
| $\Delta_{Con} (p)$  $t\left( df \right)$  $95\%CI$ | 0.09 (0.39)  0.9(148)  [-0.12, 0.3] | 0.33 (0.12)  1.6(150)  [-0.08, 0.75] |
| $\Delta_{MBSR} (p)$  $t\left( df \right)$  $95\%CI$ | -0.02 (0.84)  -0.2(151)  [-0.24, 0.2] | -0.14 (0.53)  -0.6(157)  [-0.59, 0.3] |

$\beta_{0}=\mu_{T0}\left( Con \right)$, $\beta_{1}=\mu_{T0}\left( MBSR \right)$-$\mu_{T0}\left( Con \right)$, $\beta_{2,j}=\mu_{j}\left( Con \right)$-$\mu_{T0}\left( Con \right)$,

$\beta_{3,j}=\mu_{j}\left( MBSR \right)$-$\mu_{T0}\left( MBSR \right)$-$\mu_{j}\left( Con \right)$-$\mu_{T0}\left( Con \right)$,

$\Delta_{Con}=\mu_{T12}\left( Con \right)$-$\mu_{T4}\left( Con \right)$, $\Delta_{MBSR}=\mu_{T12}\left( MBSR \right)$-$\mu_{T4}\left( MBSR \right)$,

$\bar{X_{j}}\left( MBSR \right), \bar{X_{j}}\left( Con \right)$ – sampling means
