# Supplementary material for: Pre-retirement Employees Experience Lasting Improvements in Resilience and Well-Being After Mindfulness-Based Stress Reduction
Source: Front Psychol. 2021 Jul 15;12:699088. doi: 10.3389/fpsyg.2021.699088 (PMC8321239; doi:10.3389/fpsyg.2021.699088)
Supplement: Supplementary file 4 [file Table_4.docx]

***Supplementary Material***

# Supplementary Table

**Legend, please see Excel sheet LMM.S4.xlsx**

**Supplementary Table S4. Linear mixed model analysis of ARSQ dimensions.** The table displays the measured sampling means for the 11 ARSQ dimensions for the MBSR-intervention and Control groups ($\bar{X}\left( MBSR \right)$ and $\bar{X}\left( Con \right)$, respectively) at measurements T0, T4, and T12. The LMM-estimated mean for the Control group at T0 is the regression coefficient *Beta0*, and the estimated mean difference between the MBSR and Control group is the regression coefficient *Beta1*. Regression coefficient *Beta2* is the estimated change in mean for the Control group. The LMM-estimated change in mean for the MBSR group compared with that of the Control group is the regression coefficient *Beta3*. For all tests, we report p-values (*p*), statistics (*t*), degrees of freedom (*df*), and 95% confidence intervals (95%*CI)*. The last block (T12–T4) shows sampling and estimated differences in means between the follow-up at T12 and post-intervention at T4 for either category of participants. Significant differences and p-values < 0.05 are displayed in boldface.

*Beta0* $=\mu_{T0}\left( Con \right)$, *Beta1* $=\mu_{T0}\left( MBSR \right)$-$\mu_{T0}\left( Con \right)$,

*Beta2* $=\mu_{j}\left( Con \right)$-$\mu_{T0}\left( Con \right), j=T4 or T12$ ,

*Beta3* $=\mu_{j}\left( MBSR \right)$-$\mu_{T0}\left( MBSR \right)$-$\mu_{j}\left( Con \right)$-$\mu_{T0}\left( Con \right), j=T4 or T12$ ,

$\bar{X}\left( MBSR \right), \bar{X}\left( Con \right)$ – sampling means at T0, T4, or T12
